# Improved AI-Assisted Image Recognition of Cervical Spine Vertebrae Enables Motion Pattern Analysis in Dynamic X-Ray Recordings

**DOI:** 10.3390/bioengineering13030351

**Published:** 2026-03-18

**Authors:** Esther van Santbrink, Tijmen H. W. Hijzelaar, Valérie N. E. Schuermans, Anouk Y. J. M. Smeets, Henk van Santbrink, Rob de Bie, Mitko Veta, Toon F. M. Boselie

**Affiliations:** 1 Department of Neurosurgery, Maastricht University Medical Centre, 6229 HX Maastricht, The Netherlandst.boselie@mumc.nl (T.F.M.B.); 2 Department of Neurosurgery, Zuyderland Medical Centre, 6419 PC Heerlen, The Netherlands; 3 CAPHRI Institute for Public Health and Primary Care, Maastricht University, 6211 LK Maastricht, The Netherlands; 4 Department of Biomedical Engineering, Eindhoven University of Technology, 5612 AZ Eindhoven, The Netherlands; 5 Department of Epidemiology, Maastricht University, 6211 LK Maastricht, The Netherlands

**Keywords:** cervical spine, qualitative motion analysis, image segmentation, nnU-net, X-ray recordings, motion pattern

## Abstract

Background: Qualitative motion analysis revealed that the cervical spine moves according to a consistent pattern. Current data analysis methods are limited by the extensive time required to process the retrieved data. A previous study demonstrated the feasibility of using a deep-learning model to automate analysis methods. However, segmentation accuracy needed to be improved. This study aims to improve segmentation model performance to enable reliable motion analysis. Methods: Four nnU-Net configurations were tested: baseline (A), pre-trained (B), with histogram equalization (C), and pre-trained with histogram equalization (D). Segmentation performance was evaluated using Dice Similarity Coefficient (DSC), Intersection over Union (IoU) and 95th percentile Hausdorff Distance (HD95). Vertebral rotation was estimated using mean shapes. Reliability was assessed using the Intraclass Correlation Coefficient (ICC). Sensitivity analyses were conducted. Results: Across all models, mean DSC ranged from 0.67 to 0.92, mean IoU from 0.55 to 0.85, and mean HD95 from 2.35 to 19.67 mm. After sensitivity analysis for low segmental range of motion (sROM) and low-quality recordings, the mean ICC ranged from 0.617 to 0.837 for model A, from 0.609 to 0.780 for model B, from 0.409 to 0.689 for model C, and from 0.480 to 0.835 for model D. Conclusions: This study shows that Models A and B can accurately analyze cervical motion patterns. High image contrast and an adequate sROM are essential for robust model performance. It also marks an important step toward automated qualitative motion analysis, increasing the accessibility of motion pattern evaluation.

## 1. Introduction

Qualitative motion analysis of the cervical spine revealed that a consistent motion pattern is present in 80% to 90% of healthy young individuals [1]. This pattern is defined as the order of maximum contribution of each motion segment to the extension movement. The consistent motion pattern is uncovered by the sequence of segmental contribution (SSC), which is determined by tracking the relative rotation of cervical vertebrae throughout dynamic X-ray recordings [2]. The SSC is a reliable metric, with a mean sensitivity of 90% and specificity of 85% for differentiating motion patterns between asymptomatic individuals and patients with radicular syndrome [1].

An RCT comparing anterior cervical discectomy with arthroplasty (ACDA) to anterior cervical discectomy (ACD) in patients with a radicular syndrome demonstrated that a motion pattern consistent with that of asymptomatic controls was present in 16.6% of ACDA patients before surgery. One year postoperatively, this pattern was preserved or restored in 66.7% of the ACDA group [3,4]. After a mean follow-up of 11 years, the predefined motion pattern was absent in these patients, although segmental range of motion (sROM) of the index level and upper adjacent level were maintained. The timing and underlying mechanisms responsible for this absence are unknown. In elderly individuals, quality of motion decreases over time due to the natural aging process. Qualitative analysis of cervical motion in healthy elderly individuals showed that the previously defined consistent motion pattern was absent [5]. A more comprehensive understanding how motion patterns evolve is required to define normal motion. In clinical contexts, identifying a motion pattern could influence decision-making processes, such as the selection of a surgical approach.

Qualitative motion analysis has not been widely adopted in research or clinical settings because it is labor intensive and requires trained experts. Artificial intelligence (AI) models can provide automatic and quantitative analysis of the motion pattern, thus eliminating both workload and subjectivity constraints. Several architectures for AI models have been explored to analyze medical imaging data, including 2D, 2D with temporal information (2D+t), and 3D approaches. Their segmentation performance is typically evaluated using overlap-based metrics such as the Dice Similarity Coefficient (DSC), which measures the overlap between the predicted segmentation and the ground truth. Manually annotated images are often considered the ground truth.

Previous work demonstrated the feasibility of using deep learning to analyze cervical spine motion in dynamic X-ray recordings [6]. Segmentation performance showed low to high accuracy, with Intersection over Union (IoU) ranging from 0.37 to 0.74 and DSC from 0.53 to 0.80. Reliability of motion estimation, assessed by Intraclass Correlation Coefficient (ICC), ranged from 0.62 to 0.96 for individual vertebrae and from 0.28 to 0.72 for vertebral segments, with higher scores observed in 2D+t models. These findings highlight the potential of motion pattern analysis, though segmentation performance needs to be improved to support routine application in clinical research and decision-making.

To improve segmentation accuracy, we trained a model based on nnU-Net, which is a state-of-the-art medical image segmentation model [7]. This model is trained automatically, with model training hyperparameters optimized for best performance, ensuring great efficiency. The model was originally developed using 23 biomedical segmentation datasets.

Among the different methods available to improve the performance of nnU-net, this study focuses on pre-training and histogram equalization due to their specific suitability for our dataset. Transfer learning via pre-training uses pre-trained models to mitigate data scarcity [8]. Histogram equalization augments the contrast of input images to further refine model performance [9]. This could potentially remedy the poor segmentation performance of the vertebrae that lack contrast due to overprojection of other structures, such as vertebrae C6 and C7.

This study aimed to test and improve nnU-net model performances in cervical dynamic X-ray recordings to enable reliable motion analysis in research and clinical settings.

## 2. Materials and Methods

### 2.1. Population and Manual Annotation

Previous studies by our group investigated motion patterns in healthy, asymptomatic individuals in different age groups, and in patients with a cervical radicular syndrome before and after surgery [1,3,5,10,11]. Manually annotated data from these studies were combined to train the models. Dynamic X-ray recordings were all made following the same protocol. Participants sat on a height-adjustable crutch with neck, shoulders, and head free. They moved their head fluently from full extension to full flexion and back in about 10 s, guided by a metronome. The upper body remained still throughout the movement. Shoulders and arms were kept low to ensure all cervical vertebrae remained visible during recording.

Template areas containing each vertebral body and the skull base were manually drawn by two trained individuals (TB and VS) in the median frame of the recording, labeled C0–C7. The bottom part of the sella turcica and clivus are projected in the X-ray image as a stable and hyperintense structure of the skull and serve as an ideal template to track the movement of C0 (the skull base). Image recognition software, based on matching normalized gradient field images on a best-fit principle, was used to locate the vertebrae in the other frames of the recordings [12]. The contours were manually checked and adjusted in every frame when necessary. These annotations were considered the ground truth, and previous studies have reported excellent intra- and interobserver reliability [1,5].

### 2.2. Dataset

The dataset included a total of 112 recordings, of which 89 recordings were from 39 healthy individuals and 23 recordings from preoperative patients with a cervical radicular syndrome. Recordings were captured using one of three dynamic X-ray recording systems: Siemens Arcadis Avantic VC10A (Siemens AG, Munich, Germany), Toshiba Infinix Vi (Ōtawara-shi, Tochigi-ken, Japan), or Philips Allura Xper FD20 X-ray system (Best, The Netherlands). All systems recorded at a resolution of 1024 × 1024 pixels and a frame rate of 15 frames per second. No compression was applied to the images. The recordings were split into subsets for training, validation, and testing, with a split of 60-20-20 (Table 1).

### 2.3. Development of the Model

A fully automated segmentation approach was implemented using nnU-Net for cervical vertebrae detection in dynamic X-ray recordings. nnU-Net is a self-configuring deep learning framework that automatically adapts its architecture, preprocessing, training, and postprocessing to the characteristics of a given dataset without the need for manual tuning [7]. We used the second generation of the nnU-Net model (v2), which offers improved modularity and reproducibility. In this work, the framework was used in default configuration mode, which automatically selects optimal network architecture, patch and batch sizes, and augmentation strategies. The only manual changes were the reduction in the number of training epochs, which refers to one complete pass through the entire training dataset, to 100 and the adoption of the residual encoder architecture. The standard 1000 epochs resulted in overfitting of the model and model convergence was consistently achieved within 100 epochs. The residual encoder improved segmentation performance across all model scales [13].

This architecture integrates ResNet-style blocks into the U-Net encoder to enhance feature extraction and gradient propagation. Residual skip connections allow the network to learn residual mappings, mitigating vanishing gradients and enabling deeper architectures. The nnU-Net ResEnc M variant was selected due to computational constraints.

### 2.4. Histogram Equalization

To improve anatomical visibility, histogram equalization was applied as a preprocessing step, redistributing pixel intensity values to enhance contrast and highlight structural boundaries. To avoid amplifying background noise or artifacts, binary masks isolating the patient region were generated and equalization was applied only within these regions.

**Figure 1:**
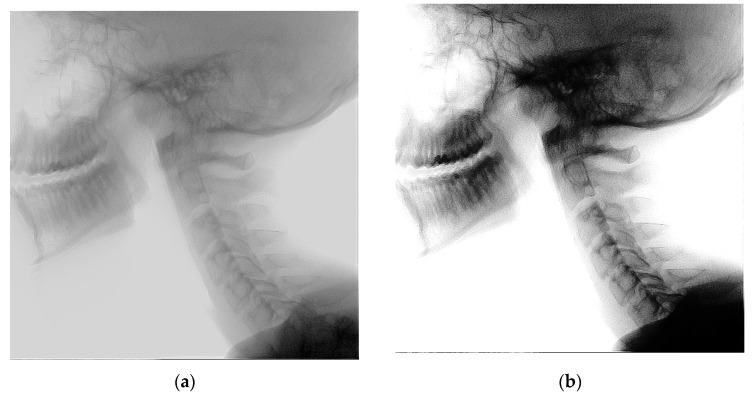

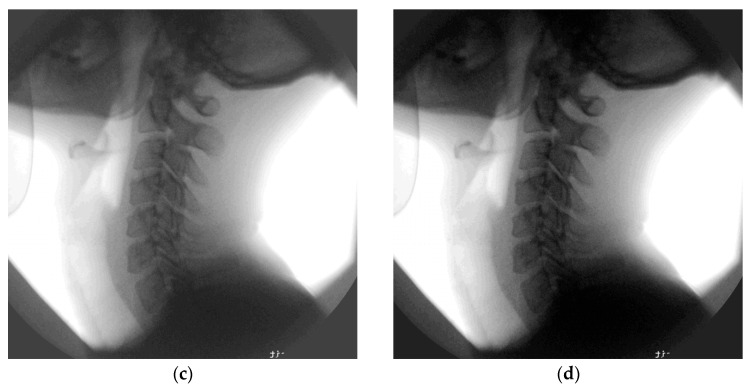
Example of histogram equalization and histogram matching. (**a**) Pre-processed image before histogram equalization; (**b**) image after histogram equalization; (**c**) pre-processed image before histogram matching; (**d**) image after histogram matching.

For recordings acquired on the Siemens systems, collimators visible in the image prevented reliable threshold-based masking of the subject. In such cases, histogram matching was performed instead of histogram equalization, aligning each sequence to a reference frame with previously demonstrated high segmentation performance [6]. In total 20 Siemens system recordings were incorporated into the dataset, accounting for 18% of the total data. The recordings were split with the same proportions among the three subsets as the global distribution. Figure 1 illustrates the differences between the image acquisition systems and provides examples of histogram equalization and histogram matching.

### 2.5. Pre-Training

Given the limited availability of annotated dynamic X-ray data, a synthetic dataset was created from the CSXA (Cervical Spine X-ray Anatomy) dataset [14]. Static cervical spine images were adapted to resemble dynamic sequences by adding noise and matching their histograms to the dynamic dataset. Each adapted image was matched to a randomly selected frame from the training or validation sets.

This synthetic dataset was used to pre-train a separate nnU-Net for 1000 epochs under default settings, except for the random rotation augmentation, which was expanded to rotate all training samples by random angles between –90° and +90°. This modification improves robustness to orientation variability, compensating for the neutral position bias of the CSXA.

The model weights were then finetuned for another 100 epochs on the target dataset. Four nnU-Net configurations were tested: baseline (A), pre-trained (B), histogram equalization (C) and pre-trained with histogram equalization (D).

### 2.6. Model Evaluation Metrics

Included evaluation metrics were Dice Similarity Coefficient (DSC), Intersection over Union (IoU), and 95th percentile Hausdorff Distance (HD95). The DSC measures the overlap between predicted (A) and ground-truth (B) segmentations, as in Equation (1).

(1) $$DSC\;(A,B)=\frac{2\times |A\cap B|}{\left|A|+|B\right|}$$

A value of 1 indicates perfect overlap and 0 indicates no overlap. While scores above 0.7 are generally considered acceptable in medical image segmentation, a stricter threshold of 0.8 was adopted in this study to ensure rotation accuracy [15].

The IoU, also known as the Jaccard Index, is defined as the ratio of the area of the intersection over the area of the union of the segmentation and the ground truth, as in Equation (2).

(2) $$IoU\;(A,B)=\frac{\left|A\cap B\right|}{\left|A\cup B\right|}$$

This measure ranges from 0 to 1 and is more sensitive to segmentation errors than Dice; here, IoU scores above 0.7 were considered sufficient [16].

The HD95 quantifies the maximum distance between the boundaries of the predicted and ground-truth segmentations, using the 95th percentile to reduce the influence of outliers, as in Equation (3)

(3) $${HD}_{95}\left(A,B\right)=max({\sup_{inf(A,B)},\sup_{inf(B,A)})}_{95}$$

Here, d(A, B) represents the Euclidean distance between boundary points. In this study, HD95 values below 16 mm, approximately the diameter of a vertebral body, were considered acceptable [17].

### 2.7. Mean Shape

In dynamic X-ray recordings, vertebrae are rigid anatomical structures and should maintain a consistent shape throughout motion. nnU-Net may produce slight variations in vertebral shape across frames due to noise, occlusion, coupled motion, or model uncertainty. To address this, a mean shape was constructed for each vertebra per recording to enforce anatomical consistency and improve reliability. The skull base (C0) was excluded due to its inconsistent visibility and variable segmentation quality across frames.

### 2.8. Outcome

The relative rotation between adjacent vertebrae was estimated using mean shapes, constructed as previously described [6]. For each frame in a sequence, the mean shape of a vertebra was translated to the centroid of the segmentation predicted by the model and iteratively rotated to achieve maximal overlap. In some cases, the previous iteration of the algorithm would find an optimum at an angle around 180 degrees higher or lower than the previous frame. To further increase the consistency of the algorithm, the range of angles was limited to ±90 degrees, which prevented these flips in subsequent frames. The fitted mean shape was used to calculate the rotation of each vertebra C in the consecutive frames of the entire recording (dθ_C_), as shown in Equation (4), where θ denotes an angle in frame t.

(4) $${d\theta }_{C(t)}=\theta_{\;C_{\left(t\right)}}-\theta_{\;C_{\left(t-1\right)}}$$

dθ_C(t)_ represents the change in absolute rotation between consecutive frames. For the first frame, dθ_C(0)_ was set to zero. With the relative rotation values of each vertebra, the relative rotation between two vertebrae (dθ_R(t)_) was calculated via Equation (5). With Ck and Cl each indicating another vertebra.

(5) $${d\theta }_{R(t)}={d\theta }_{Ck(t)}-{d\theta }_{Cl(t)}$$

To suppress high-frequency noise in the rotation signal, a low-pass Gaussian filter was applied to the rotation time series. The optimal sigma (σ) value was determined for all vertebral segments and was set to a value of 3, preserving overall motion trends while reducing noise-induced fluctuations.

### 2.9. Analyses

The Intraclass Correlation Coefficient (ICC) was used to quantify agreement between predicted and manually annotated relative rotation values. A two-way mixed-effects model with consistency agreement was applied [18]. The ICC (3, 1) model was chosen as it assesses the similarity in rotational patterns between raters instead of the absolute mean differences. ICC values above 0.6 were deemed acceptable, and those above 0.8 were considered excellent for clinical use.

Sensitivity analyses were performed to investigate the potential influence of including low-quality recordings and recordings with low sROM. Recording quality was visually assessed by two trained individuals, based on the presence of noise and overprojection. The segments that were classified as low-quality were excluded to use as a sensitivity analysis. To determine whether low sROM had an influence, vertebral segments with a ROM below 4 degrees were excluded as a sensitivity analysis.

To analyze motion patterns, the segmental rotation between each successive frame of individual motion segments from C4 to C7 was plotted against the cumulative rotation of C4 to C7. The resulting graphs were then generated and compared with the ground truth.

### 2.10. Correlation Analysis

To provide a further indication of the relation between the quality of the segmentation and the ICC of each recording, the Pearson correlation coefficient was calculated between the ICC and the mean, median and standard deviation of each segmentation metric across each recording. With these correlations, the impact of the average quality and the variability of the segmentations on the reliability of the motion pattern analysis can be determined.

## 3. Results

### 3.1. Segmentation Performance

**Figure 2:**
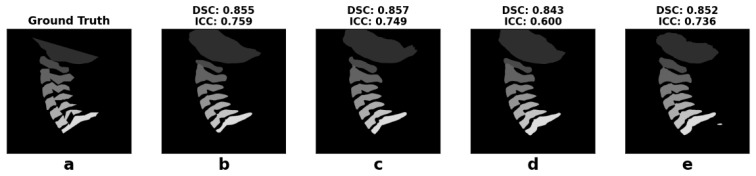
Example of segmentation performance HC02 T1e; (**a**) ground truth, (**b**) model A, (**c**) model B, (**d**) model C, (**e**) model D. The average DSC and ICC across all vertebrae of each model are displayed above every segmentation mask.

The mean DSC ranged from 0.69 to 0.91 for the baseline Model (A), from 0.71 to 0.92 for the pre-trained Model (B), from 0.69 to 0.90 for the histogram equalization Model (C), and from 0.67 to 0.91 for the pre-trained with histogram equalization Model (D). The mean IoU ranged from 0.57 to 0.84 for Model A, from 0.58 to 0.85 for Model B, from 0.57 to 0.83 for Model C, and from 0.55 to 0.84 for Model D. The mean HD95 ranged from 2.49 to 19.55 mm for Model A, from 2.35 to 19.67 mm for Model B, from 3.78 to 19.01 mm for Model C, and from 2.58 to 19.09 mm for Model D. The mean and standard deviation of the DSC, IoU, and HD95 for each vertebra across all models are presented in Table 2, calculated across all frames. Figure 2 shows an example of the segmentation performance, comparing the ground truth with the results produced by Models A–D.

### 3.2. Intraclass Correlation Coefficient

The ICC values for vertebral segments ranged from 0.449 to 0.728 for Model A, from 0.368 to 0.734 for Model B, from 0.287 to 0.612 for Model C, and from 0.382 to 0.742 for Model D, presented in Table 3. All recordings were included, except one in Model B and one in Model C, where the mean shape calculation failed, precluding the computation of rotations.

### 3.3. Sensitivity Analyses

After excluding segments due to low sROM, the ICC values ranged from 0.516 to 0.728 for Model A, from 0.399 to 0.763 for Model B, from 0.321 to 0.612 for Model C, and from 0.442 to 0.742 for Model D, presented in Table 4. After excluding segments due to low-quality, the ICC values ranged from 0.549 to 0.770 for Model A, from 0.532 to 0.761 for Model B, from 0.421 to 0.689 for Model C, and from 0.421 to 0.764 for Model D, presented in Table 5. After excluding segments due to low sROM and low-quality, the ICC values ranged from 0.617 to 0.837 for model A, from 0.609 to 0.780 for model B, from 0.409 to 0.689 for model C, and from 0.480 to 0.835 for model D, presented in Table 6. As none of the C3–C4 and C4–C5 segments had an sROM below 4 degrees, they were not filtered out, resulting in identical values in Table 3, Table 4, Table 5 and Table 6.

### 3.4. Motion Pattern Analysis

The motion pattern analyzed with Model A with the highest mean ICC is shown in Figure 3 and the motion pattern with the lowest mean ICC is shown in Figure 4. All motion patterns analyzed with Model A are presented in Appendix A (Figure A1, Figure A2, Figure A3, Figure A4, Figure A5, Figure A6, Figure A7, Figure A8, Figure A9, Figure A10, Figure A11, Figure A12, Figure A13, Figure A14, Figure A15, Figure A16, Figure A17, Figure A18, Figure A19, Figure A20 and Figure A21).

### 3.5. ICC vs. Segmentation Performance Correlation

In Table 7, the mean median and standard deviation of each segmentation metric is correlated against the ICC of each recording. None of the correlations are significant due to the low number of recordings in the dataset.

## 4. Discussion

This study aimed to test and improve nnU-net model performances on cervical dynamic X-ray recordings to support reliable motion analysis in research and clinical settings. Following sensitivity analyses, excluding low sROM and low-quality segments, the ICC values exceeded 0.6 for both the baseline Model (Model A) and the pre-trained Model (Model B), indicating that both models are suitable for motion pattern analysis.

Our findings suggest that pre-training may improve model performance, particularly for lower-quality recordings (Table 5), while the baseline model appears more effective for higher-quality recordings (Table 6 and Table 7). This implies that pre-training could be beneficial for lower-quality data, although its utility is constrained by data scarcity. Napravnik et al. evaluated pre-training across a broad range of datasets for application to more specific tasks and, consistent with our results, reported no significant improvements [19]. Closer alignment of the pre-training dataset with the downstream application may enhance model performance. In this context, pre-training on dynamic rather than static imaging could benefit the model, as the static images can only aid the model in learning vertebral shape, contrast characteristics, and general anatomy Any visual features emerging due to movement are lacking from this data. Future work may also focus on developing refined pre-training strategies that reduce or eliminate the need for human annotation [8].

Histogram equalization appeared to reduce model performance, despite visually enhancing image contrast, as seen in Figure 1. This finding was unexpected, as previous studies have reported histogram equalization to be beneficial [9]. One possible explanation is the overprojection of the shoulders over the lower vertebrae. The resulting large areas of darker pixels may skew the pixel-value histogram and interfere with the equalization process. The resulting large areas of darker pixels may skew the pixel-value histogram and interfere with the equalization process. Histogram equalization was not applied to the Siemens recordings because the visible collimators interfered with masking, yet these recordings corresponded to some of the highest model performances. The presence of collimators likely improved image contrast by focusing the beam on the central area, thereby supporting performance. Additionally, Siemens recordings lacked shoulder overprojection, which in other datasets may have been exacerbated by photon starvation, leading to magnified noise during image reconstruction [20]. Across all models, segmentation performance was generally lower for the skull base (C0) and the lower vertebrae (C6–C7). Although the skull landmark used for the ground truth is visually distinct to human observers, the resulting straight-line annotation lacks a true contrast boundary, making it more difficult for the model to predict.

**Figure A22:**
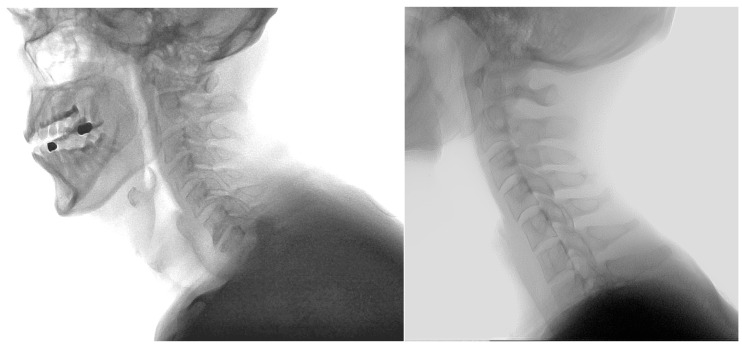
Dynamic X-ray images of the worst and best performing recordings in terms of ICC (Left: HC30 T1e, ICC 0.038. Right: HC03 T2e, ICC 0.918).

Despite Model B achieving the highest segmentation performance, with DSC rated as excellent across all levels except C0, overall consistency was limited by variability in manual annotations. The deviating morphology of the C2 vertebra contributed to inconsistent labeling, as the odontoid was included in some cases but omitted in others. Similarly, the posterior landmark of the processus spinosus was annotated inconsistently, alternating between the rounded surface and the bifid end. These inconsistencies introduced ambiguity for the model and contributed to lower ICC values for the C2–C3 segment. Segment C6–C7 also demonstrated lower ICC values. Shoulder overprojection frequently affected visualization of this segment, complicating accurate annotation for both the models but also for human observers. The relationship between segmentation performance and ICC, shown in Table 7, indicates that for most models, improved segmentation quality does not significantly increase the reliability of angle determination. However, model C has higher correlation levels than the other models, while also being the lowest performing segmentation model. This could indicate that poorer segmentation quality does impact the ICC, but with diminishing returns after a certain threshold. Sensitivity analyses demonstrated that model performance depended on recording quality, in particular high contrast and the absence of shoulder overprojection. Figure A22 highlights the contrast between the worst- and best-performing cases. While low contrast can be mitigated by increasing tube voltage, this improvement comes at the cost of higher radiation exposure, but also lower contrast in other parts of the image, for instance midcervical.

Improved ICC values observed after excluding recordings with low sROM may be explained by a lower signal-to-noise ratio in the excluded recordings. When motion amplitudes are small, the true movement between frames is similar in magnitude to imaging noise and annotation variability, causing minor segmentation inaccuracies to disproportionately affect derived relative rotation measures. The sensitivity analysis indicates that low sROM constitutes a methodological limitation for reliable motion analysis.

To mitigate large deviations in segmentation, a promptable model could offer a potential solution. A promptable model requires human input, while models with automatic inference do not. The Segment Anything Model 2 (SAM2) was also evaluated by our study team. SAM2 is a promptable segmentation model designed for both static images and video data [21]. We hypothesized that the use of manual prompts to indicate areas of interest could reduce large deviations in segmentation while substantially lowering the labor intensity of the annotation process. The segmentation and ICC results of the SAM2 model are provided in Appendix A, Table A1 and Table A2. Table A1 shows that the SAM2 segmentation results lie between those of models A and B. However, SAM2 yielded lower ICC values and required longer analysis times than the fully automated nnU-Net models. As the original SAM2 model was not trained on medical data, it may not be suitable for our dataset, even though the model was fine-tuned on dynamic X-ray recordings.

The motion pattern graphs show that, while fine details are not consistently tracked by model A, larger trends are captured well. From a biomechanical perspective, the cervical spine is not expected to exhibit continuous small oscillations during flexion and extension, which suggests that the model may, under certain conditions, provide estimates that are more physiologically plausible than the manual annotations. The lowest performing recording illustrates this limitation, as the model exhibited difficulties in reproducing rapidly changing relative rotations observed in the ground truth. Notably, this recording was also part of the poor image quality subset, which likely contributed to the reduced performance. In this case, the model’s prediction could also be more accurate than the gold standard.

Previous studies have demonstrated that motion patterns can differentiate asymptomatic individuals from patients with cervical radiculopathy [1]. Based on these observations, several clinically meaningful applications can be anticipated, particularly in the evaluation of spinal instability, the longitudinal monitoring of degenerative changes, and the assessment of post-surgical biomechanics. For example, specific motion patterns, rather than only the presence or absence of motion, may help to identify segments with a higher likelihood of heterotopic ossification, which could inform the choice between a motion-preserving implant and fusion implant. In this context, pre- and post-laminectomy analyses could offer valuable insights into how decompression alters segmental mobility and spinal alignment, which are currently assessed primarily using static imaging [22]. Likewise, longitudinal assessments of motion patterns before and after surgery may help to identify early indicators of complications such as adjacent segment disease (ASD) or post laminectomy instability.

The integration of large-scale datasets with qualitative motion analysis also holds promise for uncovering previously unrecognized kinematic patterns that may influence clinical outcomes and refine treatment strategies. Ultimately, these applications underscore the potential of AI-based motion tracking not only as a diagnostic adjunct but also as a decision-support system tailored to the individual patient. By linking qualitative motion analysis to clinical outcomes, this approach may contribute to more personalized, evidence-based management of cervical spine disorders.

## 5. Conclusions

This study demonstrates that the tested nnU-Net is capable of accurately capturing motion patterns of the cervical spine provided recording quality is sufficient and a range of motion of at least 4 degrees per vertebral segment. For potential future applications in both research and clinical practice, high image contrast and adequate sROM are recommended to ensure robust model performance. However, as these conditions may not consistently be met in routine clinical practice, broader applicability remains to be demonstrated. Moreover, in anatomically altered cases, such as patients with implants or following laminectomy, further model training will be necessary to maintain accuracy. Overall, this study provides an important step toward automated qualitative motion analysis, making the evaluation of motion patterns more accessible and scalable.

## Figures and Tables

**Figure 3:**
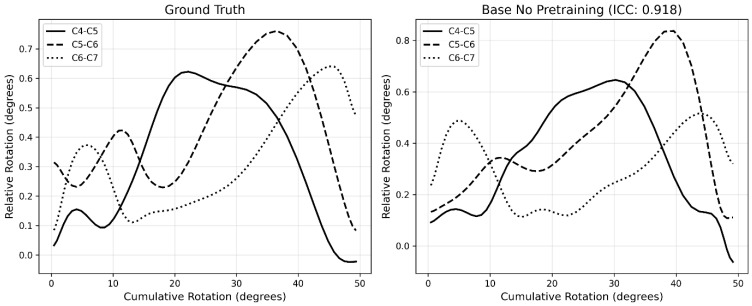
Motion pattern with the highest mean ICC (Model A, HC03 T2e ICC = 0.918). The left figure illustrates the ground truth motion pattern, whereas the right figure illustrates the motion pattern computed by the model. Extension motion over time is plotted on the x-axis, and relative rotations are plotted on the y-axis.

**Figure 4:**
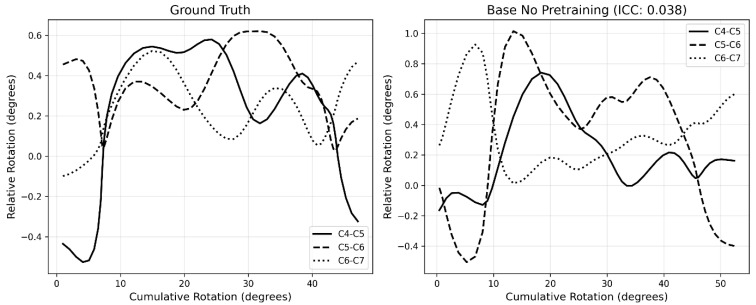
Motion pattern with the lowest mean ICC (Model A, HC30 T1e ICC = 0.038). The left figure illustrates the ground truth motion pattern, whereas the right figure illustrates the motion pattern computed by the model. Extension motion over time is plotted on the x-axis, and relative rotations are plotted on the y-axis.

**Table 1:** Overview of the included data divided into subsets, displayed for number of individuals and number of recordings.

Data Subset	Individuals (N)	Recordings (N)
Training (60%)	36	67
Validation (20%)	12	22
Testing (20%)	14	23

**Table 2:** Segmentation performance of model options A to D on the test set.

	DSC (SD)				IoU (SD)				HD95 in mm (SD)			
Model	A	B	C	D	A	B	C	D	A	B	C	D
C0	0.69 ± 0.25	0.71 ± 0.22	0.69 ± 0.24	0.68 ± 0.23	0.57 ± 0.25	0.58 ± 0.23	0.57 ± 0.25	0.55 ± 0.23	19.55 ± 16.06	19.67 ± 18.20	19.07 ± 17.94	19.25 ± 18.70
C1	0.86 ± 0.08	0.86 ± 0.08	0.85 ± 0.10	0.87 ± 0.04	0.76 ± 0.11	0.76 ± 0.10	0.74 ± 0.11	0.78 ± 0.06	4.19 ± 2.48	4.07 ± 1.55	4.74 ± 5.56	3.93 ± 1.50
C2	0.88 ± 0.06	0.89 ± 0.04	0.87 ± 0.08	0.89 ± 0.04	0.79 ± 0.09	0.80 ± 0.07	0.78 ± 0.10	0.80 ± 0.06	4.47 ± 2.04	4.15 ± 1.73	6.01 ± 10.52	4.31 ± 1.81
C3	0.90 ± 0.05	0.91 ± 0.03	0.90 ± 0.06	0.90 ± 0.03	0.82 ± 0.07	0.83 ± 0.05	0.82 ± 0.08	0.82 ± 0.05	3.19 ± 2.42	2.76 ± 1.18	3.80 ± 8.35	2.99 ± 1.68
C4	0.91 ± 0.05	0.92 ± 0.03	0.90 ± 0.06	0.91 ± 0.03	0.84 ± 0.06	0.85 ± 0.04	0.83 ± 0.08	0.84 ± 0.05	2.49 ± 1.71	2.35 ± 1.39	3.78 ± 5.85	2.58 ± 1.61
C5	0.88 ± 0.07	0.90 ± 0.04	0.87 ± 0.08	0.89 ± 0.04	0.78 ± 0.10	0.82 ± 0.06	0.77 ± 0.11	0.81 ± 0.06	4.12 ± 3.67	3.19 ± 1.83	5.30 ± 4.78	3.29 ± 1.67
C6	0.82 ± 0.15	0.90 ± 0.04	0.80 ± 0.17	0.89 ± 0.05	0.72 ± 0.18	0.82 ± 0.07	0.69 ± 0.19	0.80 ± 0.07	6.82 ± 5.92	3.68 ± 3.19	7.42 ± 6.35	4.42 ± 3.35
C7	0.75 ± 0.19	0.84 ± 0.10	0.73 ± 0.21	0.81 ± 0.13	0.63 ± 0.21	0.73 ± 0.14	0.62 ± 0.22	0.70 ± 0.16	10.06 ± 8.71	5.55 ± 4.35	11.39 ± 15.79	7.81 ± 12.39

IoU = Intersection over Union, DSC = Dice Similarity Coefficient, 95th percentile Hausdorff Distance (HD95), SD = standard deviation.

**Table 3:** ICC scores individual vertebrae calculated per model using the mean shape.

Model	A	n	B	n	C	n	D	n
Segment								
C1–C2	0.595 (−0.369–0.981)	23	0.569 (−0.423–0.966)	22	0.476 (−0.271–0.966)	22	0.577 (−0.222–0.967)	23
C2–C3	0.449 (−0.262–0.913)	23	0.546 (−0.275–0.956)	22	0.384 (−0.421–0.858)	22	0.438 (−0.386–0.920)	23
C3–C4	0.697 (−0.212–0.971)	23	0.734 (−0.041–0.967)	22	0.612 (−0.409–0.928)	22	0.724 (−0.055–0.967)	23
C4–C5	0.728 (0.086–0.961)	23	0.715 (0.080–0.965)	22	0.589 (−0.173–0.955)	22	0.742 (0.301–0.952)	23
C5–C6	0.693 (−0.042–0.968)	23	0.733 (−0.198–0.963)	22	0.342 (−0.410–0.971)	22	0.689 (−0.087–0.942)	23
C6–C7	0.449 (−0.480–0.991)	23	0.368 (−0.161–0.990)	22	0.287 (−0.151–0.990)	22	0.382 (−0.435–0.990)	23

ICC = Intraclass Correlation Coefficient.

**Table 4:** Sensitivity analysis with low sROM segments excluded. ICC values for vertebral segments calculated per model using the mean shape.

Model	A	n	B	n	C	n	D	n
Segment								
C1–C2	0.711 (−0.228–0.981)	19	0.706 (−0.423–0.966)	18	0.557 (−0.027–0.950)	18	0.720 (−0.208–0.967)	19
C2–C3	0.532 (−0.262–0.913)	20	0.583 (−0.275–0.956)	19	0.455 (−0.421–0.858)	19	0.488 (−0.386–0.920)	20
C3–C4	0.697 (−0.212–0.971)	23	0.734 (−0.041–0.967)	22	0.612 (−0.409–0.928)	22	0.724 (−0.055–0.967)	23
C4–C5	0.728 (0.086–0.961)	23	0.715 (0.080–0.965)	22	0.589 (−0.173–0.955)	22	0.742 (0.301–0.952)	23
C5–C6	0.690 (−0.042–0.968)	22	0.763 (−0.198–0.963)	21	0.330 (−0.410–0.971)	21	0.680 (−0.087–0.942)	22
C6–C7	0.516 (−0.387–0.991)	21	0.399 (−0.161–0.990)	20	0.321 (−0.151–0.968)	20	0.442 (−0.367–0.990)	21

ICC = Intraclass Correlation Coefficient.

**Table 5:** Sensitivity analysis with low-quality segments excluded. ICC values for vertebral segments calculated per model using the mean shape.

Model	A	n	B	n	C	n	D	n
Segment								
C1–C2	0.670 (−0.369–0.981)	19	0.605 (−0.375–0.966)	19	0.504 (−0.271–0.950)	18	0.638 (−0.222–0.967)	19
C2–C3	0.549 (−0.148–0.913)	19	0.597 (0.162–0.956)	19	0.498 (−0.082–0.858)	18	0.543 (−0.109–0.920)	19
C3–C4	0.770 (0.302–0.971)	19	0.761 (0.251–0.967)	19	0.689 (0.077–0.928)	18	0.764 (0.304–0.967)	19
C4–C5	0.750 (0.180–0.961)	17	0.727 (0.080–0.965)	17	0.652 (0.091–0.955)	16	0.746 (0.301–0.952)	17
C5–C6	0.715 (−0.042–0.968)	16	0.738 (−0.198–0.963)	16	0.421 (−0.318–0.971)	16	0.694 (−0.087–0.937)	16
C6–C7	0.594 (−0.043–0.991)	9	0.532 (−0.083–0.990)	9	0.427 (−0.112–0.968)	9	0.421 (−0.367–0.990)	9

ICC = Intraclass Correlation Coefficient.

**Table 6:** Sensitivity analysis with low sROM and low-quality segments excluded. ICC values for vertebral segments calculated per model using the mean shape.

Model	A	n	B	n	C	n	D	n
Segment								
C1–C2	0.837 (0.604–0.981)	15	0.780 (0.512–0.966)	15	0.617 (−0.027–0.950)	14	0.835 (0.687–0.967)	15
C2–C3	0.617 (−0.148–0.913)	17	0.636 (0.208–0.956)	17	0.561 (0.050–0.858)	16	0.594 (−0.109–0.920)	17
C3–C4	0.770 (0.302–0.971)	19	0.761 (0.251–0.967)	19	0.689 (0.077–0.928)	18	0.764 (0.304–0.967)	19
C4–C5	0.750 (0.180–0.961)	17	0.727 (0.080–0.965)	17	0.652 (0.091–0.955)	16	0.746 (0.301–0.952)	17
C5–C6	0.711 (−0.042–0.968)	15	0.743 (−0.198–0.963)	15	0.409 (−0.318–0.971)	15	0.680 (−0.087–0.937)	15
C6–C7	0.673 (0.028–0.991)	8	0.609 (−0.066–0.990)	8	0.493 (−0.112–0.968)	8	0.480 (−0.367–0.990)	8

ICC = Intraclass Correlation Coefficient.

**Table 7:** The Pearson correlation coefficient indicating the relation between segmentation performance against ICC.

Model	A		B		C		D	
Metric		n		n		n		n
Dice mean	0.047	23	0.010	22	0.654	22	0.266	23
Dice median	0.031	23	−0.081	22	0.529	22	0.028	23
Dice std	−0.027	23	−0.041	22	−0.683	22	−0.282	23
IoU mean	0.054	23	−0.008	22	0.649	22	0.253	23
IoU median	0.026	23	−0.096	22	0.528	22	0.025	23
IoU std	−0.001	23	−0.004	22	−0.674	22	−0.290	23
HD95 mean	−0.132	23	−0.209	22	−0.710	22	−0.367	23
HD95 median	−0.105	23	0.011	22	−0.600	22	−0.091	23
HD95 std	−0.128	23	−0.308	22	−0.510	22	−0.215	23

**Figure A1:**
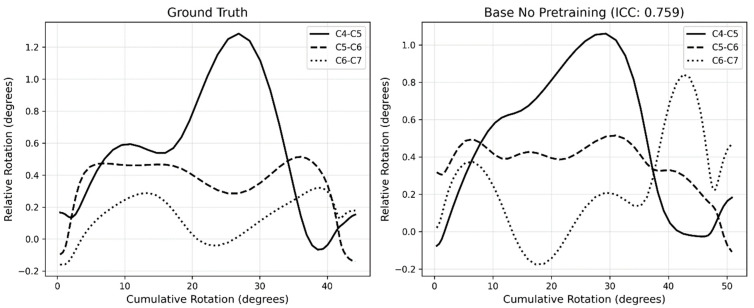
Motion pattern of HC02 T1e (Model A, ICC = 0.759).

**Figure A2:**
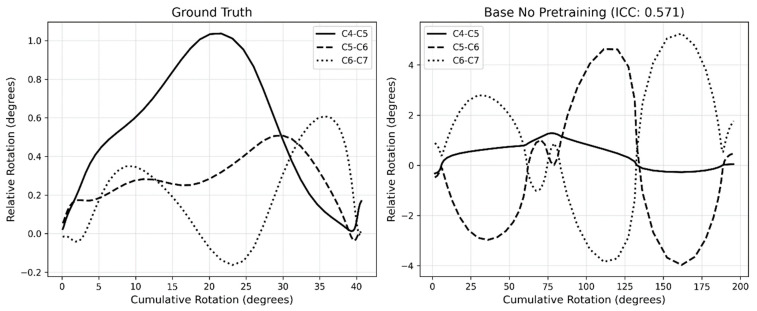
Motion pattern of HC02 T2e (Model A, ICC = 0.571).

**Figure A3:**
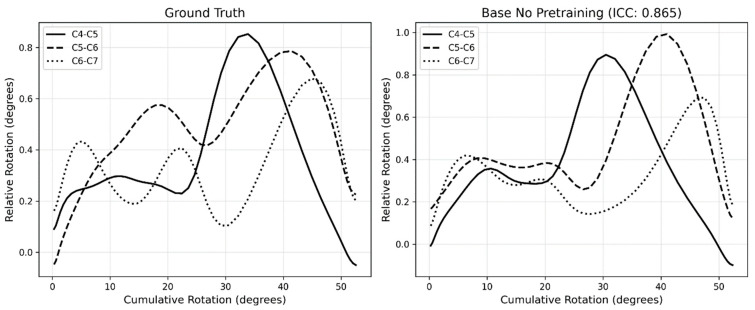
Motion pattern of HC03 T1e (Model A, ICC = 0.865).

**Figure A4:**
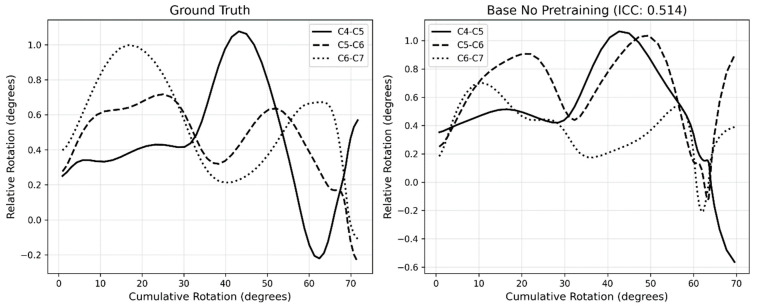
Motion pattern of HC04 T2e (Model A, ICC = 0.514).

**Figure A5:**
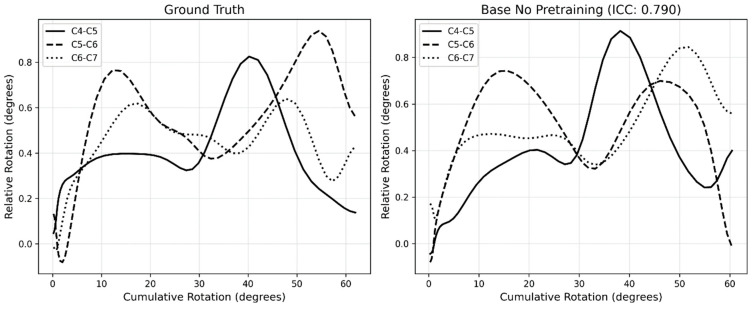
Motion pattern of HC13 T1e (Model A, ICC = 0.790).

**Figure A6:**
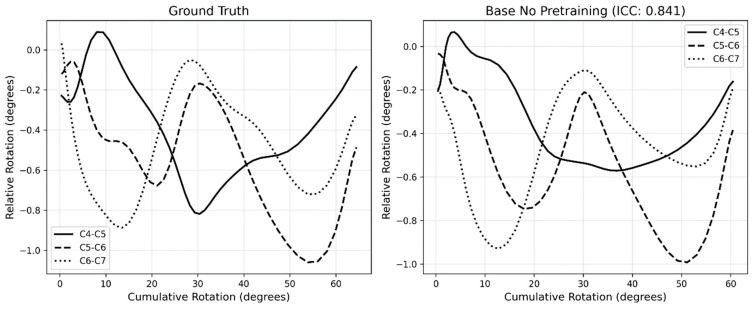
Motion pattern of HC13 T1f (Model A, ICC = 0.841).

**Figure A7:**
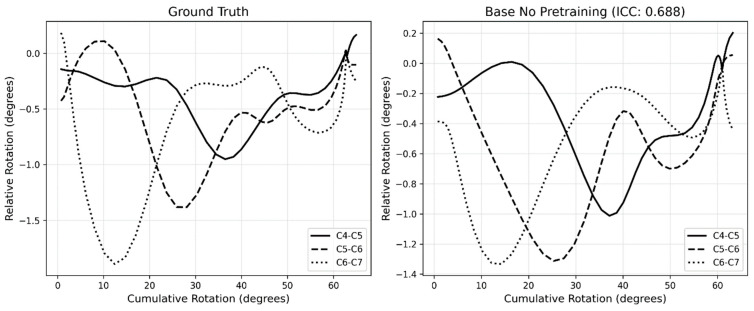
Motion pattern of HC13 T2f (Model A, ICC = 0.688).

**Figure A8:**
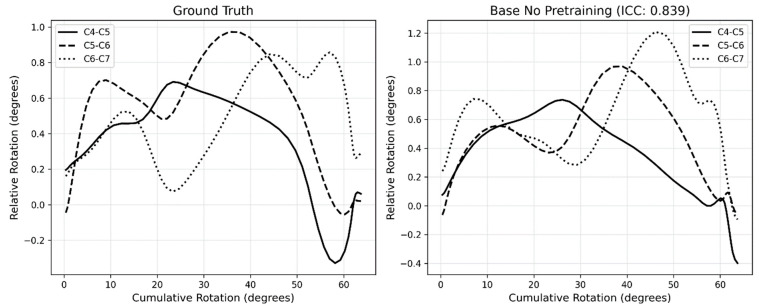
Motion pattern of HC14 T1e (Model A, ICC = 0.839).

**Figure A9:**
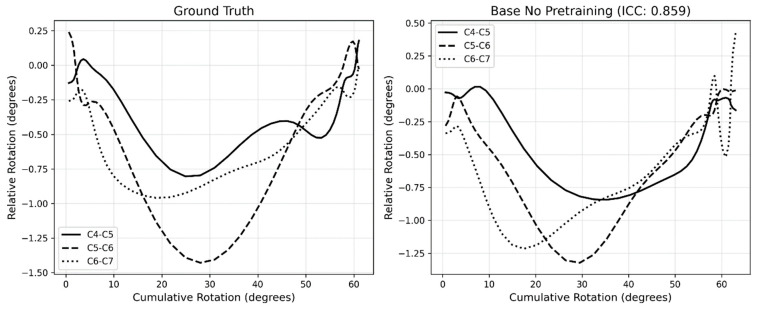
Motion pattern of HC14 T1f (Model A, ICC = 0.859).

**Figure A10:**
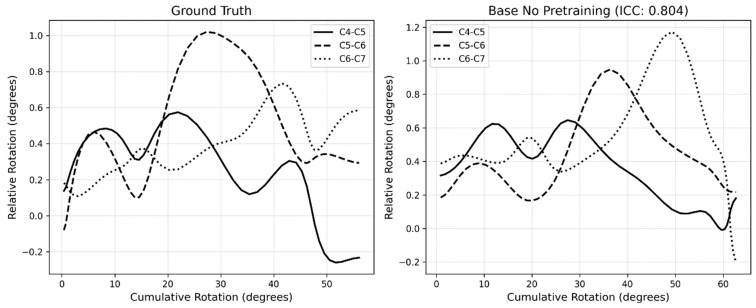
Motion pattern of HC14 T2e (Model A, ICC = 0.804).

**Figure A11:**
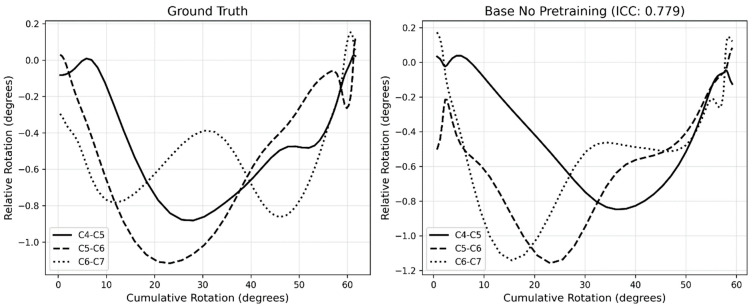
Motion pattern of HC14 T2f (Model A, ICC = 0.779).

**Figure A12:**
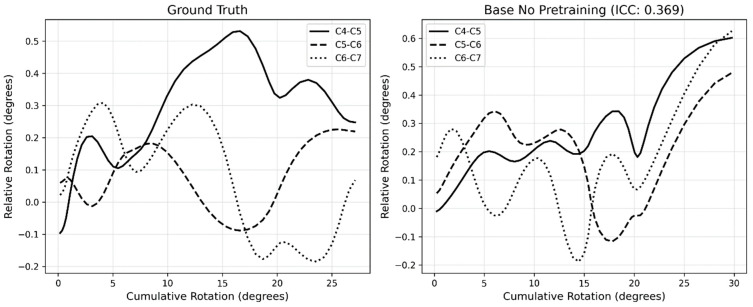
Motion pattern of HC25 T2e (Model A, ICC = 0.369).

**Figure A13:**
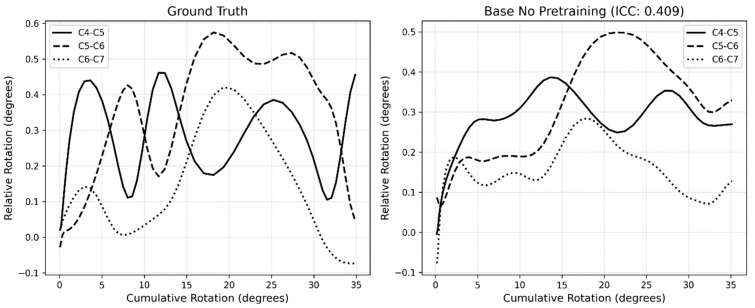
Motion pattern of HV12 T1e (Model A, ICC = 0.409).

**Figure A14:**
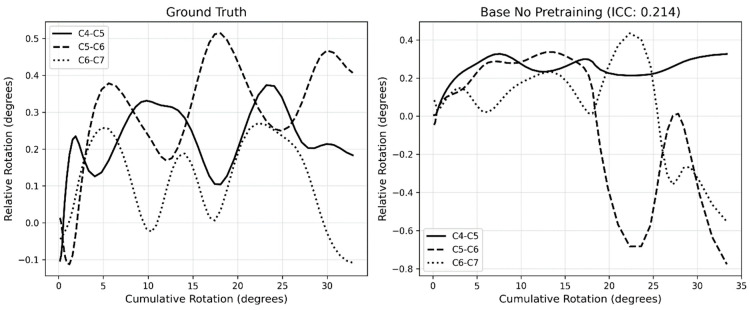
Motion pattern of HV12 T2e (Model A, ICC = 0.214).

**Figure A15:**
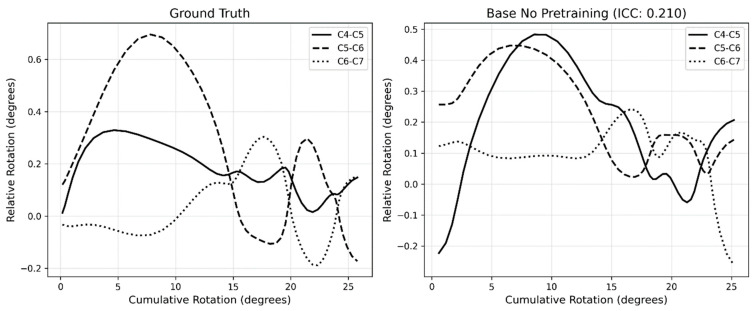
Motion pattern of HV22 T1e (Model A, ICC = 0.210).

**Figure A16:**
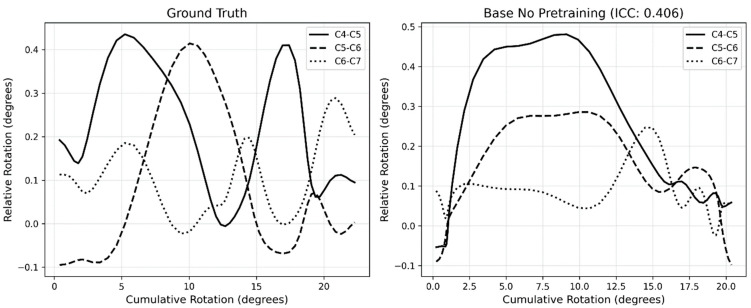
Motion pattern of HV22 T2e (Model A, ICC = 0.406).

**Figure A17:**
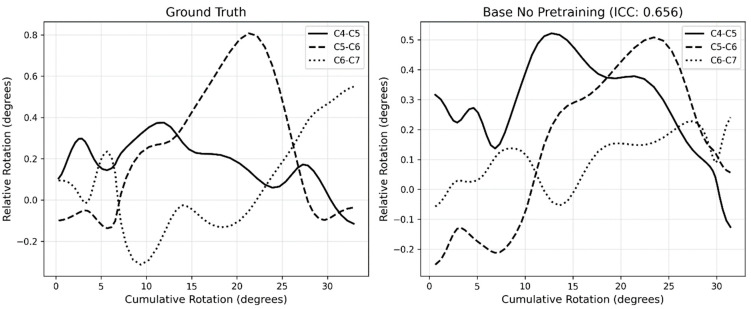
Motion pattern of HV24 T1e (Model A, ICC = 0.656).

**Figure A18:**
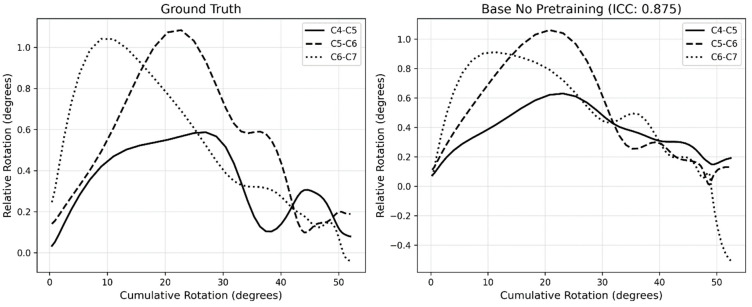
Motion pattern of P02 T1e (Model A, ICC = 0.875).

**Figure A19:**
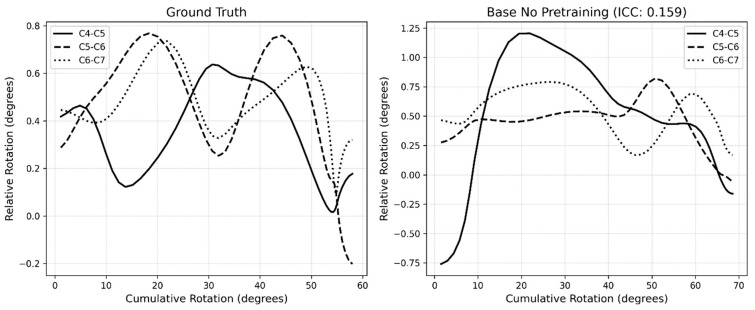
Motion pattern of P08 T1e (Model A, ICC = 0.159).

**Figure A20:**
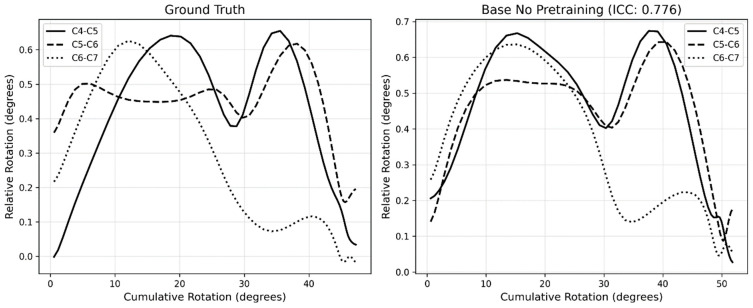
Motion pattern of P14 T1e (Model A, ICC = 0.776).

**Figure A21:**
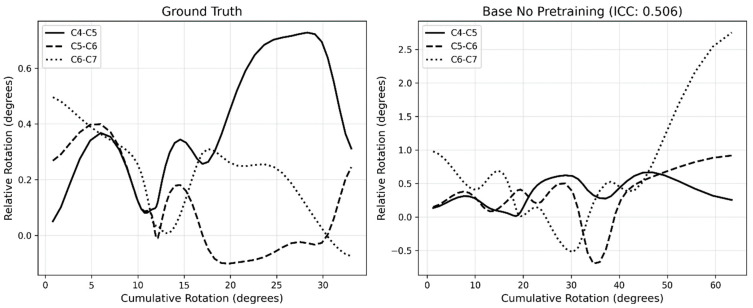
Motion pattern of P15 T1e (Model A, ICC = 0.506).

## Data Availability

The data presented in this study are available on request from the corresponding author due to privacy restrictions.

## Abbreviations

The following abbreviations are used in this manuscript:

ACD	Anterior Cervical Discectomy
ACDA	Anterior Cervical Discectomy with Arthroplasty
AI	Artificial intelligence
DSC	Dice similarity coefficient
HD95	Hausdorff Distance
ICC	Intraclass Correlation Coefficient
IoU	Intersection over Union
sROM	Segmental range of motion
SSC	Sequence of segmental contribution

## Appendix A

**Table A1:** Segmentation performance of the SAM2 model on the test set.

	DSC (SD)	IoU (SD)	HD95 in mm (SD)
C0	0.69 ± 0.25	0.57 ± 0.25	19.55 ± 16.06
C1	0.86 ± 0.08	0.76 ± 0.11	4.19 ± 2.48
C2	0.88 ± 0.06	0.79 ± 0.09	4.47 ± 2.04
C3	0.90 ± 0.05	0.82 ± 0.07	3.19 ± 2.42
C4	0.91 ± 0.05	0.84 ± 0.06	2.49 ± 1.71
C5	0.88 ± 0.07	0.78 ± 0.10	4.12 ± 3.67
C6	0.82 ± 0.15	0.72 ± 0.18	6.82 ± 5.92
C7	0.75 ± 0.19	0.63 ± 0.21	10.06 ± 8.71

IoU = Intersection over Union, DSC = Dice Similarity Coefficient, 95th percentile Hausdorff Distance (HD95), SD = standard deviation.

**Table A2:** Sensitivity analysis for the SAM2 model. ICC values for vertebral segments calculated using the mean shape algorithm.

Exclusion	None	n	Low-Quality	n	Low sROM	n	Low-Quality/sROM	n
Segment								
C1–C2	0.589 (−0.074–0.949)	23	0.625 (−0.074–0.949)	19	0.681 (0.165–0.949)	19	0.750 (0.491–0.949)	15
C2–C3	0.423 (−0.444–0.941)	23	0.491 (−0.444–0.941)	19	0.469 (−0.444–0.941)	20	0.539 (−0.444–0.941)	17
C3–C4	0.679 (0.031–0.948)	23	0.728 (0.158–0.948)	19	0.679 (0.031–0.948)	23	0.728 (0.158–0.948)	19
C4–C5	0.681 (0.123–0.963)	23	0.679 (0.123–0.963)	17	0.681 (0.123–0.963)	23	0.679 (0.123–0.963)	17
C5–C6	0.676 (−0.003–0.982)	23	0.695 (−0.003–0.982)	16	0.672 (−0.003–0.982)	22	0.689 (−0.003–0.982)	15
C6–C7	0.385 (−0.431–0.989)	23	0.522 (−0.095–0.989)	9	0.391 (−0.431–0.989)	21	0.556 (−0.095–0.989)	8

ICC = Intraclass Correlation Coefficient.
